# New Biflavonoids with α-Glucosidase and Pancreatic Lipase Inhibitory Activities from *Boesenbergia rotunda*

**DOI:** 10.3390/molecules22111862

**Published:** 2017-10-30

**Authors:** Nutputsorn Chatsumpun, Boonchoo Sritularak, Kittisak Likhitwitayawuid

**Affiliations:** Department of Pharmacognosy and Pharmaceutical Botany, Faculty of Pharmaceutical Sciences, Chulalongkorn University, Bangkok 10330, Thailand; manee_many@hotmail.com (N.C.); Boonchoo.Sr@chula.ac.th (B.S.)

**Keywords:** *Boesenbergia rotunda*, biflavonoid, flavanone-chalcone, cyclohexenyl chalcone, α-glucosidase, pancreatic lipase

## Abstract

Roots of *Boesenbergia rotunda* (L.) Mansf. are prominent ingredients in the cuisine of several Asian countries, including Thailand, Malaysia, Indonesia, India, and China. An extract prepared from the roots of this plant showed strong inhibitory activity against enzymes α-glucosidase and pancreatic lipase and was subjected to chromatographic separation to identify the active components. Three new biflavonoids of the flavanone-chalcone type (**9**, **12**, and **13**) were isolated, along with 12 known compounds. Among the 15 isolates, the three new compounds showed stronger inhibitory activity against α-glucosidase than the drug acarbose but displayed lower pancreatic lipase inhibitory effect than the drug orlistat. The results indicated the potential of *B. rotunda* roots as a functional food for controlling after-meal blood glucose levels.

## 1. Introduction

In recent years, metabolic syndrome (MetS) has gained worldwide attention due to its association with the risk of developing cardiovascular diseases and diabetes. MetS is defined as a cluster of 3 or more metabolic disorders including hypertension, obesity, high-serum triglycerides, low level of high-density lipoprotein and hyperglycemia [1,2]. Analysis of the data obtained from MetS patients has revealed the frequent co-occurrence of dyslipidemia and hyperglycemia although their cause-effect relationships are not yet understood [3]. Dietary manipulation is one of the approaches suggested for controlling MetS. Recent research has shown that botanical dietary supplements may serve as preventive agents of MetS because they usually contain structurally diverse, biologically active compounds with multiple mechanisms of action and potentially synergistic effects. Herbs with therapeutic promise for MetS have been found in various plant families, including both monocotyledons and dicotyledons, and their chemical compositions have been identified and classified as flavonoids, steroids, and triterpenoids [4]. α-glucosidase is an enzyme responsible for the breaking down of starch and disaccharides into glucose for intestinal uptake, whereas pancreatic lipase plays a key role in lipid absorption by hydrolyzing triglycerides into glycerol and free fatty acids. Plant extracts or phytochemicals that can inhibit the two enzymes are viewed as beneficial for the regulation of serum levels of sugar and fat. The present study attempted to find edible plants that may be used as functional foods in controlling postprandial hyperglycemia and hyperlipidemia.

*Boesenbergia rotunda* (L.) Mansf. (Zingiberaceae) (*B. rotunda*) is a culinary herb prominently featured in Thai cuisine, as well as in the dishes of several other Asian countries, including Malaysia, Indonesia, India, and China. The plant is commonly known as “Chinese ginger” or “fingerroot”; the latter name comes from the shape of its roots resembling fingers. In Thailand, *B*. *rotunda*, or “Krachai”, is widely cultivated for roots that are usually used as a common condiment in food such as soup or an ingredient in dipping sauce [5]. In addition, the roots are also believed to have a sexually enhancing property and often included in aphrodisiac recipes and referred to as “Thai ginseng”. Several in vivo experiments on the aphrodisiac activity of this plant have been reported [6].

*B. rotunda* has several botanical synonyms, including *Boesenbergia cochinchinensis* (Gagnep.) Loes., *Boesenbergia pandurata* (Roxb.) Schltr., *Curcuma rotunda* L., *Gastrochilus panduratus* (Roxb.) Ridl., *Gastrochilus rotundus* (L.) Alston, *Kaempferia cochinchinensis* Gagnep., *Kaempferia ovata* Roscoe, and *Kaempferia pandurata* Roxb. [7]. Previous chemical and biological studies on this plant have revealed the presence of flavonoids, styryl pyrones, and terpenoids, as well as a wide range of biological activities [8]. A recent report has suggested the potential use of this plant for the treatment of obesity [9]. During our preliminary screening of household vegetables for inhibitory activity against α-glucosidase and pancreatic lipase, we found that a methanol extract of *B. rotunda* showed strong inhibition of both enzymes (100% inhibition at 100 µg/mL). This prompted us to investigate this plant to determine the active principles, as well as their enzyme inhibition mechanisms.

## 2. Results and Discussion

### 2.1. Structure Elucidation of Bioactive Components

**Figure 1:**
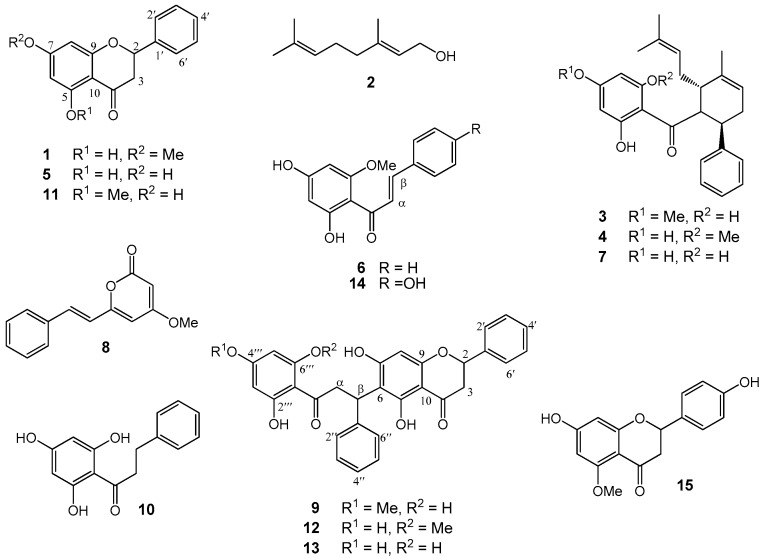
Chemical structures of Compounds **1**–**15** isolated from *Boesenbergia rotunda*.

The methanol (MeOH) extract of the roots of *Boesenbergia rotunda* was treated with CH_2_Cl_2_. The CH_2_Cl_2_ soluble fraction (50 µg/mL) exhibited strong inhibition against both α-glucosidase and lipase enzymes, but the CH_2_Cl_2_ insoluble fraction showed no activity. Separation of the CH_2_Cl_2_ extract through repeated column chromatography gave three new compounds (**9**, **12**, and **13**), along with 12 known compounds which included four flavanones (**1**, **5**, **11**, and **15**), six chalcones (**3**, **4**, **6**–**7**, **10**, and **14**), a monoterpene alcohol (**2**), and a styrylpyrone or kavalactone (**8**) (Figure 1).

Compound **9** was obtained as a pale yellow solid. The [M + Na]^+^ ion at *m*/*z* 549.1519 (calcd for C_31_H_26_O_8_Na 549.1520) in the high-resolution electrospray ionization mass spectrum (HR-ESI-MS) indicated a molecular formula of C_31_H_26_O_8_, suggesting a biflavonoid skeleton. The IR spectrum displayed bands for hydroxyl and carbonyl functionalities at 3399 and 1632 (C=O) cm^−1^, respectively, whereas the UV spectrum exhibited absorptions at 219 and 290 nm, typical of a flavanone or a dihydrochalcone structure. The COSY and HSQC spectra of **9** showed the presence of a methane-methylene coupling system (δ_H_ 5.51 (1H, dd, *J* = 13.0, 2.7 Hz, H-2)/δ_C_ 79.9 (C-2); δ_H_ 3.15 (1H, dd, *J* = 17.0, 13.0 Hz, H-3) and 2.74 (1H, dd, *J* = 17.0, 2.7 Hz, H-3)/δ_C_ 43.7 (C-3)) (Table 1) that were comparable to those observed for pinocembrin (**5**), a flavanone also isolated in this study. In addition, Compound **9** exhibited another set of aliphatic NMR resonances that could be related to those of 2′,6′-dihydroxy-4′-methoxydihydrochalcone [10], but with C-β appearing as a methine carbon (δ_H_ 4.05–4.09 (1H, m, H-α), 4.24–4.32 (1H, m, H-α)/δ_C_ 47.1; δ_H_ 5.33 (1H, t, *J* = 7.3 Hz, H-β)/δ_C_ 35.5). This was consistent with the DEPT and HSQC data of the flavanone part of **9**, which displayed C-6 as a quaternary carbon at δ 112.4. These NMR spectral properties suggested that **9** was a biflavonoid consisting of a flavanone structure connected to a dihydrochalcone unit through a C–C bond between C-6 and C-β. The HMBC correlation of H-β (δ 5.33) to C-5 (δ 163.0) and C=O (δ 205.6) confirmed the existence and position of this bridge. In addition, the MeO group at δ 3.77 should be placed at the *para*-position due to its NOESY correlation peak with the two aromatic protons at δ 5.98. Based on the above-mentioned spectral analyses, **9** was determined to be a biflavonoid derived from flavanone-chalcone coupling, with the structure as shown (Figure 1), and was given the trivial name rotundaflavanochalcone. Compound **9** is the first member of this class of dimeric flavonoids.

Compound **12** was isolated as a light brown solid. The ion [M + Na]^+^ at *m*/*z* 549.1527 (calcd for C_31_H_26_O_8_Na 549.1520) in the HR-ESI-MS indicated a molecular formula of C_31_H_26_O_8_, which was identical with that of **9**. In addition, **12** showed IR bands at 3391 (OH), 2918 (C–H), and 1631 (C=O) cm^−1^ and UV absorptions at 220 and 292 (4.33) nm that were similar to those of **9**. These implied that **12** was an isomer of **9**. Although ^1^H- and ^13^C-NMR signals for the flavanone part of **12** were nearly superimposed with those of **9** (Table 1), the resonances for the dihydrochalcone unit were slightly different. In **12**, the acetate-derived aromatic ring of the dihydrochalcone moiety was not symmetrically substituted, as indicated from the distinct chemical shifts observed for H-3′′′/C-3′′′ (δ_H_ 5.92 (d, *J =* 2.2 Hz)/δ_C_ 96.8) and H-5′′′/C-5′′′ (δ_H_ 6.03 (br s)/δ_C_ 91.8). The MeO group (δ_H_ 3.85 (3H, s)/δ_C_ 56.2) in **12** was located at C-6′′′, as established by the NOESY correlation peak between the MeO protons and H-5′′′ (δ 6.03). The HMBC of **12** showed ^3^*J*_CH_ couplings between H-β (δ 5.26) and C-5 (δ 162.9) and therefore confirmed the C-6 to C-β linkage for the flavanone-dihydrochalcone union. Thus, it was concluded **12** was a structural isomer of **9** with the structure as shown, and the compound was named *iso*-rotundaflavanochalcone.

Compound **13** was purified as a light brown solid. It displayed IR bands for OH (3398 cm^−1^) and C=O (1632 cm^−1^) functionalities, as well as UV absorptions for a flavanone/dihydrochalcone structure (204 and 291 nm). The HR-ESI-MS data displayed [M + Na]^+^ at *m*/*z* 535.1365 (calcd for C_30_H_24_O_8_Na 535.1363), corresponding to the molecular formula C_30_H_24_O_8_. The ^1^H- and ^13^C-NMR data of **13** showed close resemblance to that of **9**, suggesting a similar flavanone-dihydrochalcone skeleton (Table 1). The only exception was the absence of NMR signals for a MeO group in **13**, consistent with its molecular mass being 14 amu lower than that of **9**. The long-range coupling observed for H-β (δ 5.33) and C-5 (δ 163.0) in the HMBC spectrum confirmed the connection of C-6 of the flavanone part to C-β of the dihydrochalcone unit. Based on the above spectroscopic evidence, **13** was determined to be the de-*O*-methyl derivative of **9**, and therefore named de-*O*-methyl rotundaflavanochalcone.

The unique structures of the cyclohexenyl chalcones (**3**, **4**, and **7**) and the flavonochalcones (**9**, **12**, and **13**) deserve some discussion on their biogenesis. Both groups can be considered as products of the Michael addition reaction of the chalcone skeleton with a corresponding nucleophile. The former group involves a geranyl-derived diene as the nucleophile attacking the α,β-unsaturated carbonyl, whereas the latter engages a flavanone molecule as the Michael donor.

**Figure 2:**
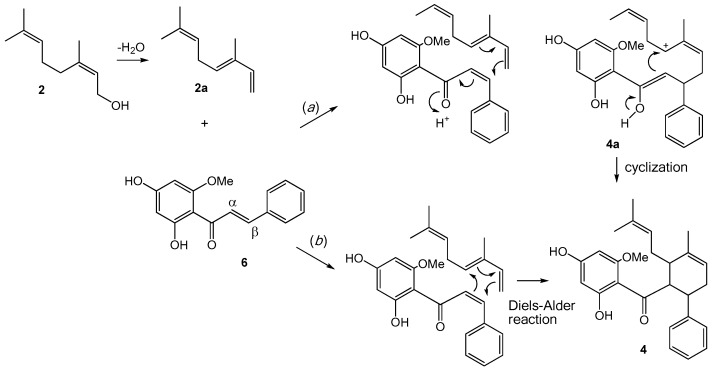
Proposed biogenetic pathway for cyclohexenyl chalcones **3**, **4**, and **7**.

The cyclohexenyl chalcones **3**, **4**, and **7** can be viewed as produced from the prenylation, or more specifically, the geranylation of a chalcone (Figure 2). For example, the biogenesis of **4** can begin with the Michael addition of the chalcone cardamonin (**6**) with **2a**, a putative dehydration product of geraniol (**2**). This then leads to the formation of structure **4a** (path *a*), which can subsequently cyclize to give isopanduratin A (**4**), a cyclohexenyl chalcone. Alternatively, **4** may be formed directly from the [4 + 2] addition between **2a** and **6** via Diels-Alder reaction (path *b*). Either way, the α,β-unsaturated carbonyl of the chalcone skeleton is the key starting structure.

**Figure 3:**
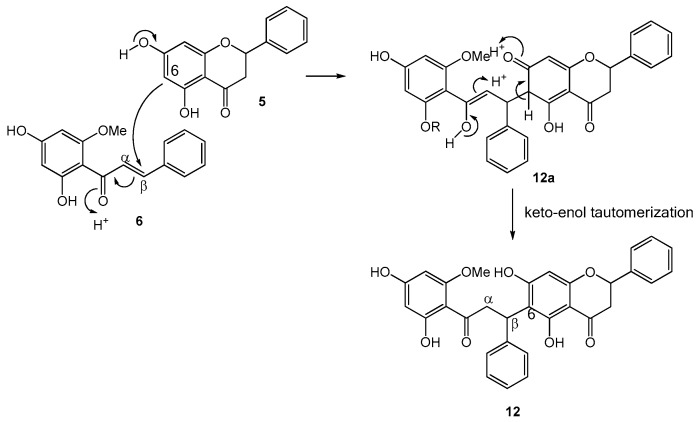
Proposed biogenetic pathway for flavanone-coupled chalcones **9**, **12**, and **13**.

An analogous biogenetic pathway can be proposed for the biflavonoids **9**, **12**, and **13** (Figure 3). For instance, the biogenesis of **12** can be initiated by the nucleophilic attack from C-6 of the flavanone pinocembrin (**5**) onto C-β of the chalcone cardamonin (**6**) to form structure **12a**. Through keto-enol tautomerization, **12a** isomerizes to generate *iso*-rotundaflavanochalcone (**12**). To the best of our knowledge, **9**, **12**, and **13** are the first representatives of biflavonoids with C-6 (flavanone) to C-β (chalcone) linkage. So far, there has been only one other example of flavanone-chalcone-derived biflavonoid, which, however, involves a different type of coupling [11].

### 2.2. α-Glucosidase Inhibitory Activity

Table 2 shows α-glucosidase inhibitory activity of Compounds **1**–**15**. Only the cyclohexenyl chalcones (**3**–**4**, **7**) and the biflavonoids (**9**, **12**–**14**) displayed significant activity (>90% inhibition at 20 µg/mL), whereas the other compounds were devoid of activity. The flavanone-coupled chalcones **9**, **12**, and **13** (IC_50_ 1.3–3.4 µM) possessed about 3–10 times higher activity than the prenylated chalcones **3**, **4**, and **7** (IC_50_ 4.6–12.7 µM). It should be noted that both groups of compounds were much stronger α-glucosidase inhibitors than the drug acarbose (IC_50_ 1.2 mM).

**Figure 4:**
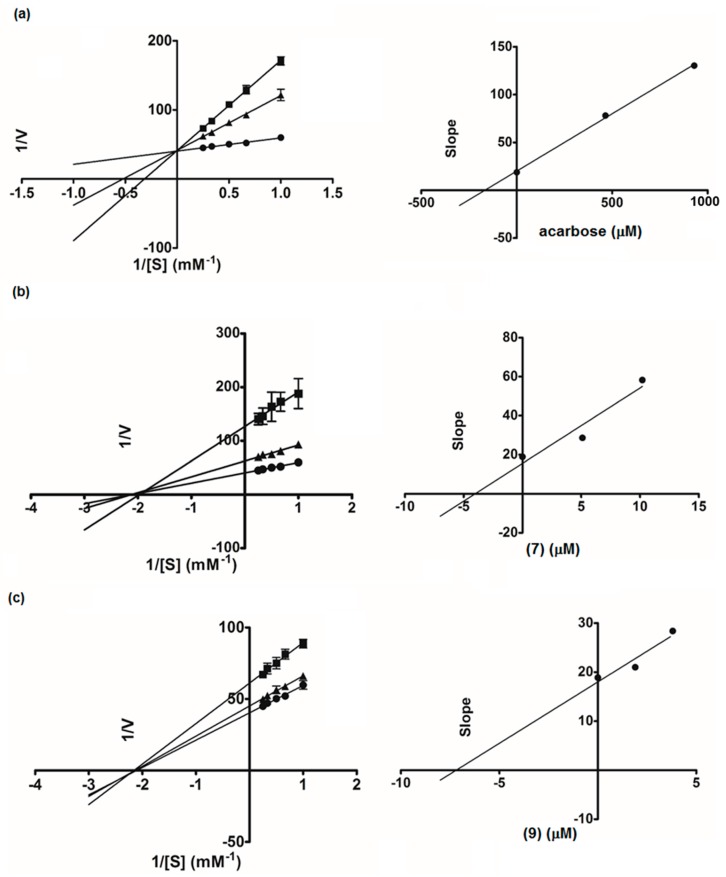
Lineweaver-Burk plots of acarbose (**a**): ● control, ■ acarbose 600 µg/mL, ▲ acarbose 300 µg/mL; hydroxypandurantin A (**7**) (**b**): ● control, ■ (**7**) 4 µg/mL, ▲ (**7**) 2 µg/mL; Compound **9** (**c**): ● control, ■ (**9**) 2 µg/mL, ▲ (**9**) 1 µg/mL. The secondary plot of each compound is on the right.

To further investigate the inhibitory characteristics of these active compounds, we conducted kinetics studies using Lineweaver-Burk plots of the reciprocal of velocity (1/V) against the reciprocal of substrate concentration (1/[S]) (Figure 4). Hydroxypanduratin A (**7**) and rotundaflavanochalcone (**9**) were selected as the representatives of the cyclohexenyl chalcones and the dimeric flavanone-chalcones, respectively, for enzyme kinetics analysis. The Lineweaver-Burk plots of **7** and **9** produced straight lines with intersections on the *x*-axis (the Michaelis-Menten constant, K*_m_* 0.46 and 0.47 mM, respectively), indicating a non-competitive mode of inhibition. Acarbose, as expected, showed the competitive type of inhibition, with an intersection of the data lines on the *y*-axis. A secondary plot of each compound was then constructed to calculate the inhibition constant (K*_i_*). We found that both **7** (K*_i_* 4.03 µM) and **9** (K*_i_* 7.19 µM) showed much higher affinity to the enzyme than acarbose (K*_i_* 167.27 µM). All these results suggested the possibility that these structurally complex chalcones (**3**, **4**, **7**, **9**, **12**, and **13**) could be employed as good α-glucosidase inhibitors of plant origin. It should be noted that a large number of dietary flavonoids and polyphenols with α-glucosidase inhibitory activity have been reported [12], and recently, their synergistic effects with acarbose have been demonstrated [13].

### 2.3. Lipase Inhibitory Activity

**Figure 5:**
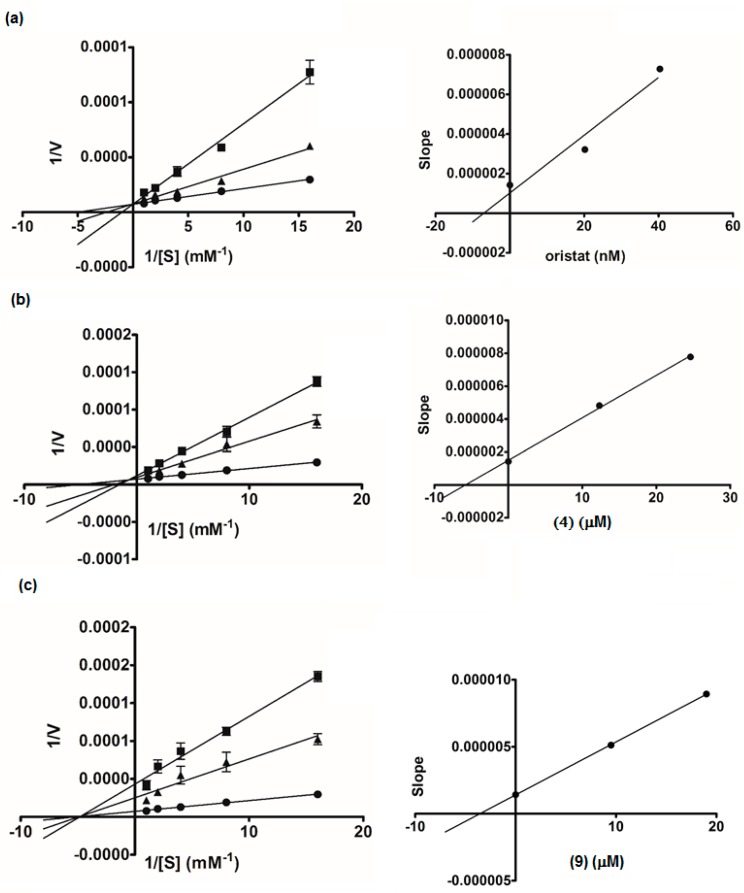
Lineweaver-Burk plots of orlistat (**a**): ● control, ■ orlistat 20 ng/mL, ▲ orlistat 10 ng/mL; isopanduratin A (**4**) (**b**): ● control, ■ (**4**) 10 µg/mL, ▲ (**4**) 5 µg/mL; Compound **9** (**c**): ● control, ■ (**9**) 20 µg/mL, ▲ (**9**) 10 µg/mL. The secondary plot of each compound is on the right.

Compounds **1**–**15** were evaluated for lipase inhibitory activity in comparison with the drug orlistat (Table 2). The low percentage of inhibition and high IC_50_ values of these compounds indicated that all are not potent inhibitors of lipase. However, all the prenylated chalcones and the dimeric flavanone-chalcones (**3**, **4**, **9**, **12**, and **13**), except for **7**, displayed recognizable activity. Due to the limited amounts of the isolates, only **4** and **9** (selected as representatives of the cyclohexenyl chalcones and the flavanone-coupled chalcones, respectively) were subjected to kinetics assays (Figure 5). A Lineweaver-Burk plot of orlistat showed the competitive type of inhibition, with all the data lines crossing the *y*-axis. The plot for **9** displayed *x*-intercept (K*_m_* 0.20 mM), indicating the non-competitive mode of inhibition, but the lines obtained for **4** did not intersect either the *x*- or *y*-axis, suggesting that **4** was a mixed-type inhibitor. The secondary plots generated for **4**, **9**, and orlistat revealed that the K*_i_* values of **4** and **9** (5.79 and 3.55 μM, respectively) were far higher than that of orlistat (7.18 nM). These data implied that these chalcones (**3**, **4**, **9**, **12**, and **13**) had low affinity to the lipase enzyme.

### 2.4. Therapeutic Implications

A recent clinical study has shown that α-glucosidase inhibitor drugs (α-GIs) can effectively control blood glucose and body weight in obese type 2 diabetic patients [14]. Thus, the potent α-GIs identified in *B. rotunda* roots, i.e., the prenylated (**3**, **4**, and **7**) and the flavanone-coupled (**9**, **12**, and **13**) chalcones, are good lead molecules or candidates for the development of functional foods for people that are prone to diabetes. Although these chalcones appear to be weak lipase inhibitors, they may well have the possibility of exhibiting anti-obesity activity through other mechanisms. For example, panduratin A (**4**) has been shown to attenuate high-fat-diet (HFD)-induced obesity in mice by activating AMP-activated protein kinase (AMPK), and this leads to the increase of fatty acid oxidation and inhibition of lipid synthesis [9,15].

Ethnomedicinal data show that people have consumed roots of *B. rotunda* as food for centuries without reports of toxicity [7,8]. Recently, the safety of ingestion of *B. rotunda* roots has been scientifically proven by several in vivo experiments. Biochemical, hematological, and histological parameters obtained for animals fed with *B. rotunda* extracts showed no signs of adverse effects [16,17,18]. Based on these toxicity studies, occasional consumption of *B*. *rotunda* roots extract should be harmless, although the safety of long-term use awaits further study.

## 3. Materials and Methods

### 3.1. General Experimental Procedures

Optical rotations were obtained with a Perkin-Elmer 341 polarimeter (Boston, MA, USA). UV spectra were measured on a Milton Roy Spectronic 300 Array spectrophotometer, and IR was recorded on a Perkin-Elmer FT-IR 1760x spectrophotometer (Boston, MA, USA). High-resolution electrospray ionization mass spectra (HR-ESI-MS) were recorded with a Bruker micro TOF mass spectrometer (Billerica, MA, USA). NMR spectra were obtained with a Bruker Avance DPX-300 FT-NMR spectrometer (Billerica, MA, USA). Vacuum liquid chromatography (VLC) and column chromatography (CC) were performed on silica gel 60 (Merck, 70–230 µm, Darmstadt, Germany), silica gel 60 (Merck, 230–400 nm) or Sephadex LH-20 (Pharmacia, Piscataway, NJ, USA). Yeast α-glucosidase enzyme, *p*-nitrophenol-α-d-glucopyranoside, pancreatic lipase, and 4-methylumbelliferyl oleate were purchased from Sigma Chemical, Inc. (St. Louis, MO, USA), and acarbose from Fluka Chemical (Buchs, Switzerland).

### 3.2. Plant Materials

*B. rotunda* roots were purchased from a local fresh market in Bangkok, Thailand. Voucher specimens (KL-03-2559) have been on deposit at the Department of Pharmacognosy and Pharmaceutical Botany, Chulalongkorn University, Bangkok, Thailand.

### 3.3. Extraction, Isolation, and Purification

Dried roots of *B. rotunda* (900 g) were ground and macerated with MeOH at room temperature (3 × 6 L). The filtrate was then concentrated under vacuum to give 84 g of crude extract. The extract was then treated with CH_2_Cl_2_ (3 × 200 mL) to give CH_2_Cl_2_ soluble (71 g) and insoluble (13 g) fractions. When evaluated at 50 µg/mL, the CH_2_Cl_2_ soluble fraction exhibited about 98% inhibition of enzymes α-glucosidase and pancreatic lipase, whereas the CH_2_Cl_2_ insoluble fraction did not show such activity (<50% inhibition). The CH_2_Cl_2_ soluble fraction was then subjected to vacuum liquid column chromatography (VLC) on silica gel, eluted with hexane/ethyl acetate (EtOAc)/MeOH in a polarity-gradient manner to yield 15 fractions (F1–F15). Fraction 3 (22 g) was recrystallized from a mixture of hexane–EtOAc to give colorless crystals of pinostrobin (**1**, 11 g). Fraction 5 (6.6 g) was chromatographed on Sephadex LH-20 (MeOH) to give 12 fractions (F5A–F5L). Fraction F5C (772 mg) was separated on silica gel with hexane–EtOAc gradient elution to yield a yellow oil of geraniol (**2**, 565 mg). Fraction F5G (2.2 g) was separated by silica gel column chromatography (CC) with hexane–CH_2_Cl_2_ polarity gradient elution to give panduratin A (**3**, 419 mg). Fraction 6 (9 g) was subjected to CC on silica gel (hexane–CH_2_Cl_2_ polarity gradient elution) to give 13 fractions (F6A–F6M). Fraction 6H (600 mg) was further purified by Sephadex LH-20 (MeOH) to furnish isopanduratin A (**4**, 282 mg). Fraction 6J (3.5 g) was recrystallized from a hexane–EtOAc mixture to give pinocembrin (**5**, 2.7 g). Fraction 7 (2.9 g) was separated by CC on silica gel (polarity gradient elution with hexane–CH_2_Cl_2_) to give 18 fractions (F7A–F6R). Fraction 7Q (169 mg) was further purified by Sephadex LH-20 (MeOH) to give cardamonin (**6**, 45 mg). Fraction 9 (3.7 g) was separated on Sephadex LH-20 (MeOH) to furnish 7 fractions (F9A–F9G). Fraction 9B (1.3 g) was subjected to CC on silica gel (polarity gradient elution with hexane–EtOAc) to give 21 fractions (F9B1–F9B21). Fraction 9B7 (507 mg) was further separated by Sephadex LH-20 (MeOH) to give hydroxypanduratin A (**7**, 24 mg) and crude **8**, which was repurified on a silica gel column (hexane–CH_2_Cl_2_ polarity gradient elution) to give 5,6-dehydrokawain (**8**, 79 mg). Fraction 10 (729 mg) was rechromatographed on Sephadex LH-20 (MeOH) to give 11 fractions (F10A–F10K). Fraction 10J, after removal of the solvent, gave Compound **9** (33 mg). Fraction 10F (63 mg) was subjected to CC on silica gel with CH_2_Cl_2_-EtOAc polarity gradient elution to give 2′,4′,6′-trihydroxydihydrochalcone (**10**, 24 mg). Fraction 13 (3.7 g) was rechromatographed on Sephadex LH-20 (MeOH) to give 10 fractions (F13A–F13J). Fraction 13C (805 mg), after drying, was recrystallized from a CH_2_Cl_2_-MeOH mixture to give alpinetin (**11**, 219 mg). Fraction 13F (267 mg) was subjected to CC on silica gel (hexane–EtOAc polarity gradient) to give 16 fractions (F13F1–F13F16). After evaporation of the solvent, Fraction 13F6 gave Compound **12** (21 mg), and Fraction 13I yielded **13** (12 mg). Further purification of Fraction 13F8 (56 mg) by CC on silica gel with CH_2_Cl_2_-EtOAc polarity gradient elution furnished 4,4′,6′-trihydroxy-2′-methoxychalcone (**14**, 4 mg). Fraction 14 (1.4 g) was separated on a Sephadex LH-20 (MeOH) column to give 9 fractions (F14A–F14I). Fraction 14I (214 mg) was purified by CC (silica gel/CH_2_Cl_2_–EtOAc polarity gradient elution) to give 7, 4′-dihydroxy-5-methoxyflavanone (**15**, 42 mg).

*Pinostrobin* (**1**): white solid; ^1^H- and ^13^C-NMR data were identical with reported values [19]; HR-ESI-MS *m*/*z* 293.0781 [M + Na]^+^ (calcd for C_16_H_14_O_4_Na 293.0784).

*Geraniol* (**2**): pale yellow oil; ^1^H- and ^13^C-NMR data were in agreement with reported values [20]; HR-ESI-MS *m*/*z* 177.1249 [M + Na]^+^ (calcd for C_10_H_8_ONa 177.1250).

*Panduratin A* (**3**): yellow solid; ^1^H- and ^13^C-NMR data were superimposable with literature values [21]; HR-ESI-MS *m*/*z* 429.2032 [M + Na]^+^ (calcd for C_26_H_30_O_4_Na 429.2036).

*Isopanduratin A* (**4**): yellow solid; ^1^H- and ^13^C-NMR data were identical with published values [22]; HR-ESI-MS *m*/*z* 429.2038 [M + Na]^+^ (calcd for C_26_H_30_O_4_Na 429.2036).

*Pinocembrin* (**5**): white solid; ^1^H- and ^13^C-NMR data were in agreement with literature values [19]; HR-ESI-MS *m*/*z* 279.0627 [M + Na]^+^ (calcd for C_15_H_12_O_4_Na 279.0628).

*Cardamonin* (**6**): yellow solid; ^1^H- and ^13^C-NMR data matched literature values [23]; (HR-ESI-EMS *m*/*z* 271.0969 [M + H]^+^ (calcd for C_16_H_15_O_4_ 271.0965).

*Hydroxypanduratin A* (**7**): yellow solid; ^1^H- and ^13^C-NMR data agreed with literature values [24]; HR-ESI-MS *m*/*z* 415.1876 [M + Na]^+^.(calcd for C_25_H_28_O_4_Na 415.1880).

*5,6*-*Dehydrokawain* (**8**): pale yellow solid; ^1^H- and ^13^C-NMR data agreed with reported values [23,25]; HR-ESI-MS *m*/*z* 229.0852 [M + H]^+^ (calcd for C_14_H_13_O_3_ 229.0859).

*Compound* (**9**): yellow solid; ${\left[\alpha \right]}_{D}^{25}$ −4.1 (*c* 0.001, MeOH); UV (MeOH) λ_max_ (log ε): 219 (4.44), 290 (4.33) nm; IR (CHCl_3_) ν_max_ 3399 (OH), 2918 (C–H), 1632 (C=O), cm^−1^; ^1^H- and ^13^C-NMR data see Table 1; HR-ESI-MS *m*/*z* 549.1519 [M + Na]^+^ (calcd for C_31_H_26_O_8_Na 549.1520).

*2′,4′,6′*-*Trihydroxydihydrochalcone* (**10**): yellow solid;^1^H- and ^13^C-NMR data were identical with reported values [26]; HR-ESI-MS *m*/*z* 259.0965 [M + H]^+^ (calcd for C_15_H_15_O_4_ 259.0965).

*Alpinetin* (**11**): white solid; ^1^H- and ^13^C-NMR data were superimposable with previously described values [19]; HR-ESI-MS *m*/*z* 271.0965 [M + H]^+^ (calcd for C_16_H_15_O_4_ 271.0965).

*Compound* (**12**): light brown solid; ${\left[\alpha \right]}_{D}^{25}$ −5.5 (*c* 0.001, MeOH); UV (MeOH) λ _max_ (log ε): 220 (4.39), 292 (4.33) nm; IR (CHCl_3_) ν_max_ 3391 (OH), 2918 (C–H), 1631 (C=O) cm^−1^; ^1^H and ^13^C-NMR data see Table 1; HR-ESI-MS *m*/*z* 549.1527 [M + Na]^+^ (calcd for C_31_H_26_O_8_Na 549.1520).

*Compound* (**13**): light brown solid; ${\left[\alpha \right]}_{D}^{25}$ −6.0 (*c* 0.001, MeOH); UV (MeOH) λ_max_ (log ε): 204 (4.04), 219 (4.37), 291 (4.28) nm; IR (CHCl_3_) ν_max_ −3398 (OH), 2918 (C–H), 1632 (C=O) cm^−1^; ^1^H- and ^13^C-NMR data see Table 1; HR-ESI-MS *m*/*z* 535.1365 [M + Na]^+^ (calcd for C_30_H_24_O_8_Na 535.1363).

*Helichrysetin* (**14**): yellow solid; ^1^H- and ^13^C-NMR data were identical with reported values [27]; HR-ESI-MS *m*/*z* 309.0739 [M + Na]^+^ (calcd for C_16_H_14_O_5_Na 309.0733).

*4′*-*7*-*Dihydroxy*-*5*-*methoxyflavanone* (**15**): pale yellow solid; ^1^H- and ^13^C-NMR data were in agreement with reported values [28,29]; HR-ESI-MS *m*/*z* 309.0720 [M + Na]^+^ (calcd for C_16_H_14_O_5_Na 309.0733).

### 3.4. Assays for α-Glucosidase Inhibitory Activity

The assay was based on the capacity of the sample to inhibit the hydrolysis of *p*-nitrophenyl-α-d-glucoside (PNPG) by α-glucosidase to release *p*-nitrophenol (PNP), a yellow color agent that can be monitored at 405 nm [30]. Briefly, 10 μL of sample solution and 40 μL of 0.1 unit/mL α-glucosidase were incubated at 37 °C for 10 min. Then, 50 μL of 2 mM PNPG was added, and the mixture was further incubated at 37 °C for 20 min. One hundred microliters of 1 mM Na_2_CO_3_ was added, and the progress of the enzyme inhibition was monitored by measuring the absorbance at 405 nm. Acarbose was used as a positive control.

### 3.5. Evaluation of Lipase Inhibitory Activity

The lipase inhibitory activity was assessed by measuring the amount of 4-methylumbelliferone (4MU), using 4-methylumbelliferyl oleate (4MUO) as substrate. The assay was modified from a previously reported method [31]. In brief, 25 μL of a blank, plant extract vehicle, or a sample solution and 50 μL of 0.5 mM 4MUO in buffer consisting of 13 mM Tris-HCl, 150 mM NaCl, and 1.3 mM CaCl_2_ (pH 8.0), were mixed in 96-well microtiter plate. Twenty-five microliters of lipase from porcine pancreas (Type II), prepared at 0.5 mg/mL in the same buffer, was then added to start the enzyme reaction. After incubation at room temperature for 30 min, 100 μL of 0.1 M sodium citrate (pH 4.2) was added to stop the reaction. Fluorescence, corresponding to released 4MU, was then measured with excitation and emission wavelengths of 355 and 460 nm, respectively. Orlistat was used as a positive control.

### 3.6. Kinetic Study of α-Glucosidase and Lipase Inhibition

An enzyme kinetic analysis was performed based on the α-glucosidase or lipase inhibition assay as described above. The PNPG concentrations varied from 1 to 4 mM, and the 4-MUO concentrations varied from 0.063 to 1 mM in the absence or presence of the test compounds for kinetic assays of α-glucosidase and lipase, respectively. The inhibition mode was determined by double-reciprocal Lineweaver-Burk plot (1/V vs. 1/[S]). In order to estimate the K*_i_* value, slopes of double-reciprocal lines were used to construct a secondary plot, and K*_i_* was calculated from the line equation of the plot [32].

## 4. Conclusions

The present study investigated *B. rotunda* roots for chemical components with inhibitory activity against enzymes α-glucosidase and pancreatic lipase. A total of 15 compounds (**1**–**15**) were isolated and structurally determined. Three of the isolates (**9**, **12**, and **13**) are new compounds and form a novel class of biflavonoids. Six compounds (**3**, **4**, **7**, **9**, **12**, and **13**) showed stronger inhibitory activity and higher affinity to α-glucosidase than the drug acarbose. The flavanone-coupled chalcones (**9**, **12**, and **13**) had higher activity than the prenylated chalcones (**3**, **4**, and **7**). Both groups exhibited a non-competitive mode of inhibition against α-glucosidase. Some of the compounds isolated from *B. rotunda* presented recognizable inhibitory effects against lipase. Taken all together, the above findings indicate that *B. rotunda* roots are potential functional food for the prevention or management of metabolic disorders, such as diabetes and obesity.

## Figures and Tables

**Table 1:** NMR data of Compounds **9**, **12**, and **13** (δ in ppm and *J* in Hz). ^a–c^

Position	9		12		13	
	δ_H_	δ_C_	δ_H_	δ_C_	δ_H_	δ_C_
*Flavanone unit*						
2	5.51 dd (13, 2.7)	79.9	5.52 dd (13, 2.8)	79.9	5.52 dd (13, 2.4)	79.9
3	2.74 dd (17, 2.7)	43.7	2.75 dd (17, 2.8)	43.7	2.75 dd (17, 2.4)	43.7
	3.15 dd (17, 13)		3.15 dd (17, 13)		3.15 dd (17, 13)	
	2.74 dd (17, 2.7)		2.75 dd, (17, 2.8)		2.75 dd, (17, 2.4)	
4		197.1		197.1		197.1
5		163.0		162.9		163.0
6		112.4		112.5		112.5
7		162.0		164.5		162.0
8	6.04 s	95.7	6.04 s	95.7	6.04 s	95.7
9		165.2		162.0		165.0
10		103.1		103.1		103.2
1		140.1		140.1		140.1
2′(6′)	7.08–7.55 m	127.3	7.06–7.56 m	127.3	7.06–7.56 m	127.3
3′(5′)	7.08–7.55 m	129.4	7.06–7.56 m	128.8	7.06–7.56 m	128.8
4′	7.08–7.55 m	129.4	7.06–7.56 m	126.3	7.06–7.56 m	129.4
*Dihydrochalcone unit*						
1′′		145.6		145.5		145.7
2′′(6′′)	7.08–7.55 m	128.7	7.06–7.56 m	129.4	7.06–7.56 m	129.4
3′′(5′′)	7.08–7.55 m	128.5	7.06–7.56 m	128.5	7.06–7.56 m	128.5
4′′	7.08–7.55 m	126.2	7.06–7.56 m	129.4	7.06–7.56 m	126.2
1′′′		105.9		106.0		105.4
2′′′		165.2		168.2		165.2
3′′′	5.98 s	94.3	5.92 d (2.2)	96.8	5.92 s	95.8
4′′′		166.6		165.4		165.4
5′′′	5.98 s	94.3	6.03 br s	91.8	5.92 s	95.8
6′′′		165.2		165.1		165.2
C=O		205.6		205.2		205.2
α	4.05–4.09 m	47.1	3.80–3.99 m	47.3	3.98–4.09 m	47.0
	4.24–4.32 m		4.07–4.19 m		4.22–4.30 m	
β	5.33 t (7.3)	35.5	5.26 t (7.2)	35.8	5.33 t (7.3)	35.5
HO-5	12.75 s	-	12.76 s	-	12.73 s	-
MeO-4′′′	3.77 s	55.7	-	-		-
MeO-6′′′	-	-	3.85 s	56.2		-

^a^ Measured in acetone-*d*_6_; ^b^ Assignments were based on DEPT, HSQC, HMBC, ^1^H-^1^H COSY, and ^1^H-^1^H NOESY experiments; ^c^ m: signal multiplicity pattern is unclear.

**Table 2:** α-Glucosidase and inhibitory activities of Compounds **1**–**15**.

Compound	α-Glucosidase		Lipase	
	% Inhibition (at 20 µg/mL)	IC_50_ (µM)	% Inhibition (at 20 µg/mL)	IC_50_ (µM)
**1**	10.0 ± 2.9	-	7.9 ± 0.8	-
**2**	9.6 ± 4.0	-	30.6 ± 2.0	-
**3**	94.7 ± 0.4	12.7 ± 1.3	72.3 ± 0.1	17.1 ± 3.7
**4**	97.8 ± 0.8	7.5 ± 0.6	75.2 ± 1.1	15.1 ± 3.3
**5**	19.8 ± 0.1	-	3.7 ± 1.3	-
**6**	19.7 ± 0.9	-	7.7 ± 1.9	-
**7**	98.5 ± 0.4	4.6 ± 0.4	49.3 ± 2.7	-
**8**	9.5 ± 2.5	-	33.7 ± 1.0	-
**9**	100.0 ± 0.0	2.4 ± 0.4	69.1 ± 1.6	25.8 ± 2.6
**10**	88.1 ± 0.9	32.0 ± 2.2	48.7 ± 3.5	-
**11**	7.0 ± 2.0	-	8.0 ± 1.9	-
**12**	100.0 ± 0.2	3.4 ± 0.9	67.8 ± 1.3	30.1 ± 2.3
**13**	100.0 ± 0.1	1.3 ± 0.2	80.5 ± 0.6	10.6 ± 1.2
**14**	97.1 ± 0.5	-	70.9 ± 2.0	-
**15**	20.9 ± 2.1	-	44.7 ± 1.7	-
**Acarbose**	-	1155.5 ± 23.0		
**Orlistat**			-	31.4 ± 0.6 nM
